# Local and bulk ^13^C hyperpolarization in nitrogen-vacancy-centred diamonds at variable fields and orientations

**DOI:** 10.1038/ncomms9456

**Published:** 2015-09-25

**Authors:** Gonzalo A. Álvarez, Christian O. Bretschneider, Ran Fischer, Paz London, Hisao Kanda, Shinobu Onoda, Junichi Isoya, David Gershoni, Lucio Frydman

**Affiliations:** 1 Department of Chemical Physics, Weizmann Institute of Science, Rehovot 76100, Israel; 2 Department of Physics, Technion, Israel Institute of Technology, Haifa 32000, Israel; 3 National Institute for Materials Science, 1-1 Namiki, Tsukuba, Ibaraki 305-0044, Japan; 4 Japan Atomic Energy Agency, 1233 Watanuki, Takasaki, Gunma 370-1292, Japan; 5 Research Center for Knowledge Communities, University of Tsukuba, 1-2 Kasuga, Tsukuba, Ibaraki 305-8550, Japan

## Abstract

Polarizing nuclear spins is of fundamental importance in biology, chemistry and physics. Methods for hyperpolarizing ^13^C nuclei from free electrons in bulk usually demand operation at cryogenic temperatures. Room temperature approaches targeting diamonds with nitrogen-vacancy centres could alleviate this need; however, hitherto proposed strategies lack generality as they demand stringent conditions on the strength and/or alignment of the magnetic field. We report here an approach for achieving efficient electron-^13^C spin-alignment transfers, compatible with a broad range of magnetic field strengths and field orientations with respect to the diamond crystal. This versatility results from combining coherent microwave- and incoherent laser-induced transitions between selected energy states of the coupled electron–nuclear spin manifold. ^13^C-detected nuclear magnetic resonance experiments demonstrate that this hyperpolarization can be transferred via first-shell or via distant ^13^Cs throughout the nuclear bulk ensemble. This method opens new perspectives for applications of diamond nitrogen-vacancy centres in nuclear magnetic resonance, and in quantum information processing.

## Abstract (web summary)

Hyperpolarization of nuclear spins for enhancing the sensitivity of magnetic resonance can typically be achieved at low temperatures. Here, the authors demonstrate room-temperature polarization of ^13^C derived from optically pumped electrons of nitrogen vacancies in diamonds with arbitrary orientations.

## Graphical abstract

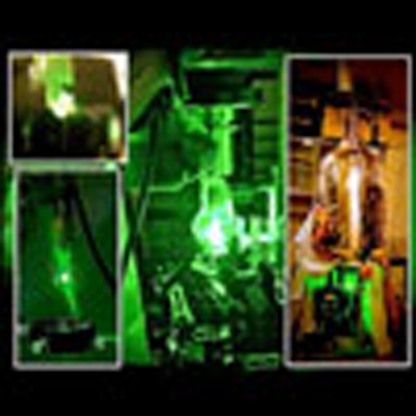

Nuclear spins are central actors in nuclear magnetic resonance (NMR) spectroscopy and imaging (MRI), providing important information at the molecular, micro- and mesoscopic levels for a wide variety of physical, chemical and biological processes^1^,^2^. Owing to their long relaxation times, nuclear spins are also promising vehicles for storing and manipulating quantum information, making them useful resources for potential quantum devices that extend the limits of classical computers^3^,^4^,^5^. Many of these uses derive from the weakness of nuclear spin interactions, making NMR methods remarkably noninvasive but also the observation of NMR signals challenging and characterized by intrinsically low signal-to-noise ratios. Dynamic nuclear polarization^6^,^7^,^8^ can bypass these limitations by transferring spin polarization from electrons to nuclei; for this process to be even more efficient cryogenic temperature operations are usually required^9^,^10^,^11^, leading to nuclear polarization enhancements encompassing several orders of magnitude. Electronic spins in nitrogen-vacancy (NV) centres are also promising alternatives for polarizing nuclear spins at room temperature conditions^12^,^13^,^14^,^15^,^16^,^17^,^18^,^19^. Hitherto proposed electron-to-nuclear polarization transfer methods, however, have so far demanded finely tuned energy-matching conditions, both in terms of field strength and of its orientation with respect to the diamond crystal to perform efficiently. These prerequisites pose an obstacle for utilizing these methods in generic applications, including the use of diamond powders as tracers in biological MRI injection studies^20^,^21^,^22^, or as sources to hyperpolarize nuclei outside the diamond in NMR-based analyses^23^. It is hereby shown that these demands for achieving a robust electron**-**to**-**nuclear polarization transfer can be relaxed by the inclusion of incoherent processes^24^,^25^. The method hereby proposed includes a continuous microwave (MW) irradiation that coherently addresses the 0↔−1 (or +1) *S*=1 electronic spin transition while exploiting the asymmetry of the electron–nucleus hyperfine (HF) interaction imposed by the spin-1 nature of the NV centre^26^,^27^,^28^,^29^. This selective MW addressing is combined with the incoherent spin repopulation effects introduced by the optical pumping process^30^,^31^ to produce an imbalance between the populations of the coupled nuclear spins. This achievement of steady-state ^13^C polarization is here demonstrated over a range of magnetic field strengths and orientations as well as of electron–nuclear HF interactions, both in optical studies of single NV centres and in shuttled NMR measurements of bulk samples. Moreover, by exploiting the versatility of this new approach, light is shed on the role played by HF-imposed spin–diffusion barriers in the achievement of bulk nuclear spin hyperpolarization.

## Results

### The MW-driven-relaxation-aided polarization transfer scheme

**Figure 1:**
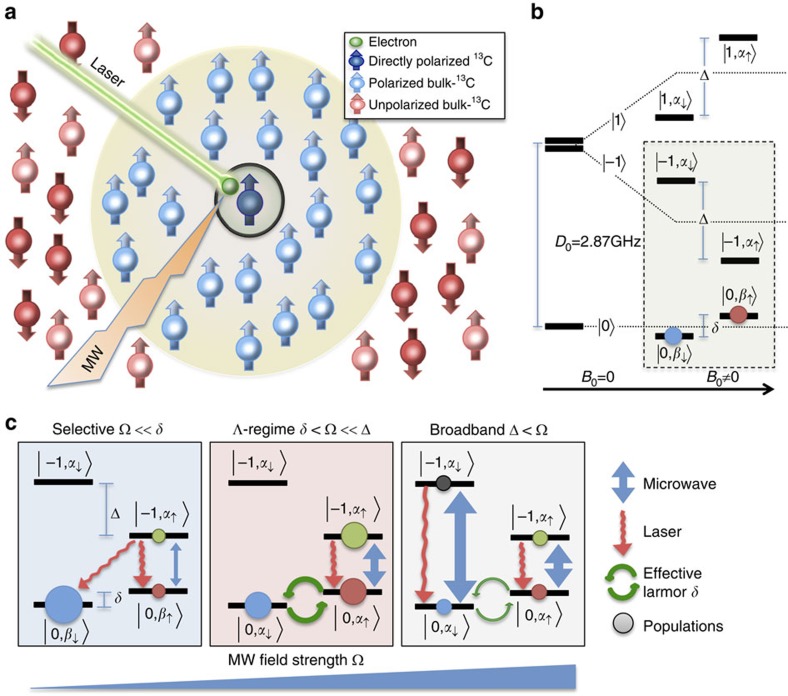
MW-driven ^13^C polarization derived from optically pumped nitrogen-vacancy centres. (**a**) Spin-1 NV electronic defect strongly coupled to a ^13^C nucleus (black circle), irradiated simultaneously by MW (orange lightning bolt) and laser fields (green beam). The optically pumped NV-spin (green sphere) transfers its polarization to the coupled nucleus (dark blue sphere) and eventually to the remaining ^13^C (the bulk) by interactions within a dipolar spin network (light blue spheres). The red spheres are unpolarized nuclei. (**b**) Energy-level diagram of the electron defect, hyperfine coupled to a ^13^C in presence of a potential magnetic field *B*_0_ (without MW fields). The dashed box shows the energy levels addressed, and stresses an initial state containing equal populations on the lower 
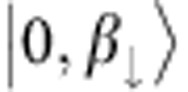
, 
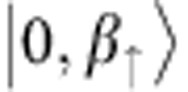
 eigenstates after optical pumping. (**c**) Spin dynamical regimes determined by the relation between the MW power and the energy splittings. The solid black lines only represent the eigenstates 
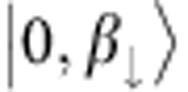
, 
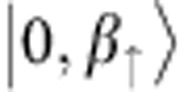
, 
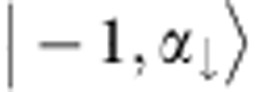
 and 
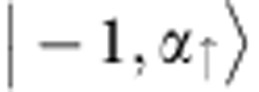
 of the system in the selective regime; in the remaining cases these lines represent states 
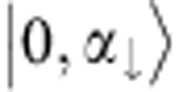
, 
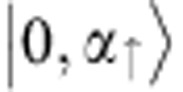
, 
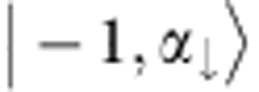
 and 
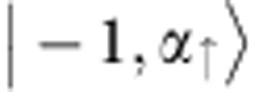
 that are relevant for the MW selection rules, but where 
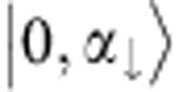
 and 
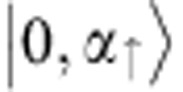
 are linear superpositions of the eigenstates 
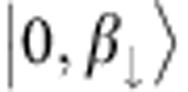
 and 
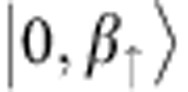
. Blue vertical arrows represent the MW excitation on resonance with the transition 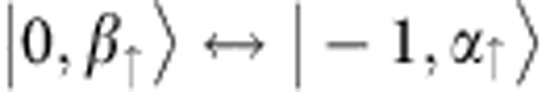, circular green arrows represent an effective Larmor precession with frequency *δ*, while curly red arrows represent a laser-induced relaxation-like process conserving the nuclear spin state but driving the incoherent 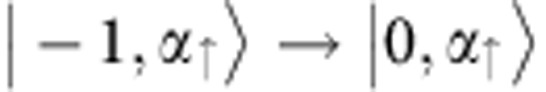 optical pumping^52^. The filled and coloured circles schematize the populations of each state resulting from these dynamics. Although not explicitly shown in these energy-level diagrams, the electronic and nuclear spins involved in these manifolds are also coupled by dipole–dipole interactions to the ^13^C ensemble via a spin-coupling network, enabling further polarization transfers to the bulk (**a**).

Figure 1a defines the relevant system and active interactions that will be used to introduce our polarization transfer proposal. The system involves a single NV *S*=1 spin exhibiting a ground-state zero-field splitting *D*_0_=2.87 GHz. This electronic spin is coupled to nearby or distant ^13^C nuclear spins (*I*=1/2) through HF interactions and can be optically pumped to populate the electronic *m*_*s*_=0 level^30^,^31^. The application of a weak static magnetic field 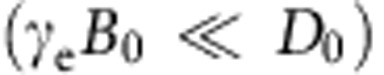 induces an additional splitting of the electronic/nuclear states, leading to the energy-level diagram in Fig. 1b. Our scenario assumes a selective MW irradiation that solely addresses the electron 
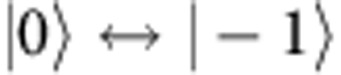
 spin transitions, allowing one to treat the system as a four-level manifold (Fig. 1b). The nuclear spin components of the eigenstates associated with *m*_s_=0 (
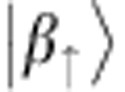
 and 
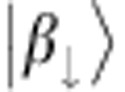
) and *m*_s_=−1 (
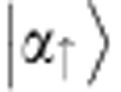
 and 
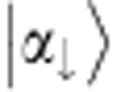
) have different quantization axes because of the asymmetry imposed by a HF interaction that is absent if the electron state is *m*_s_=0 and present if *m*_s_=−1 (refs 26, 27, 28, 29). As further discussed in Methods and Supplementary Note 1, the corresponding eigenenergies exhibit splittings *δ* and *Δ* for the 0 and −1 manifolds, respectively. Notice that this asymmetry is due to the *S*=1 nature of the NV centre, and not due to particular details of couplings within the sample. These differences in level splitting and in quantization axes among nuclear spins that are initially unpolarized are here exploited to hyperpolarize them.

Different polarization transfer routes can be activated by irradiating this HF-coupled system. We discriminate these depending on the relative MW field strength Ω *vis-a-vis* the energy splittings *δ* and *Δ*, determining which eigenstates will participate in the dynamics. In the selective 
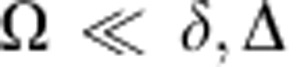
 regime (Fig. 1c), the MW-induced transitions involve solely two directly addressed eigenstates. Therefore, the population of a third, non-addressed state associated with the *m*_s_=0 manifold grows systematically as driven by the laser-induced relaxation processes. This generates an imbalance between the 
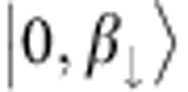
 and 
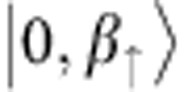
 populations, equivalent to a nuclear polarization whose direction is defined by the *m*_s_=0 eigenstate manifold. As the MW power increases, a Λ-regime where transitions are induced among three eigenstates^29^ is reached (Fig. 1c). We describe this 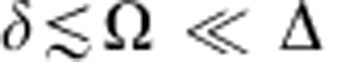 regime in a basis set 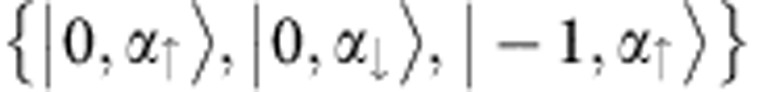; here 
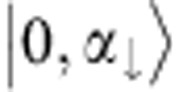
 is a dark state for the MW in the sense that 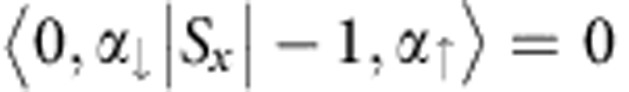, and 
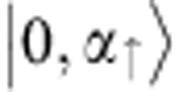
 is a bright state 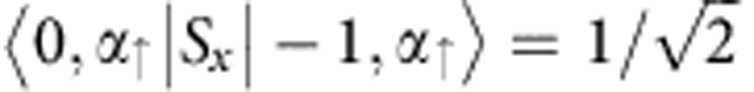 that is addressed by the MW. Owing to the different HF properties associated with *m*_s_=0 and *m*_s_=−1, 
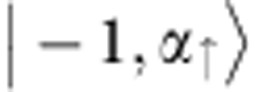
 is an eigenstate but 
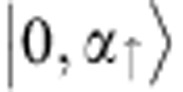
 and 
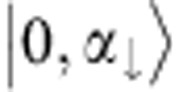
 are not, and therefore they oscillate at an effective nuclear Larmor frequency *δ*. As further detailed in Methods and in Supplementary Note 1, this precession takes place around an effective field defined by the quantization axis of the 
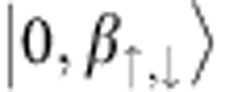
 states. This field is given by the nuclear Zeeman axis if the non-secular terms of the HF coupling can be disregarded; if this is not possible, a second-order perturbation approach can be used to account for the Hamiltonian determining the effective precession field (see Supplementary Figs 1–4). The combined action of this nuclear precession, the MW irradiation and the laser-driven relaxation results in a redistribution of the initial populations over the three-level system, biasing the bright nuclear-state population, over its dark counterpart. For the example in Fig. 1c, a net nuclear magnetization pointing along the 
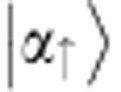
 quantization axis, defined at weak fields by the spin's HF coupling to *m*_s_=−1, is then obtained. Finally, as the MW power is further increased, a broadband regime where the electron state is flipped without regards to the nuclear spin state is reached; in such an instance no nuclear polarization enrichment is predicted.

### Nuclear hyperpolarization in the vicinity of the NV centre

**Figure 2:**
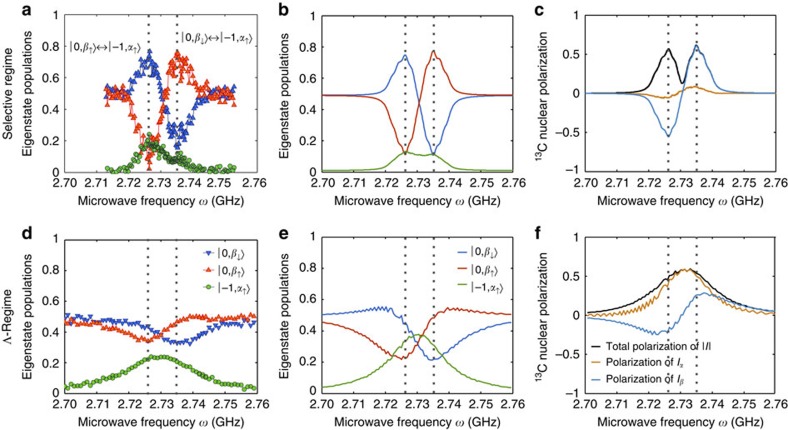
Hyperpolarization of a first-shell nuclear spin using a MW-driven, optically pumped nitrogen-vacancy centre. Experiments were conducted by orienting a magnetic field 
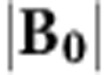
=4.04 mT at *θ*≈42° and *φ*≈85° polar and azimuthal angles with respect to the zero-field tensor **D**_**0**_ of the NV centre. The effective ^13^C Larmor is then *δ*≈8.8 MHz, while the first-shell hyperfine interaction is Δ≈130 MHz. Two MW power strengths were calibrated corresponding to Rabi frequencies of Ω≈1.4 MHz<8.8 MHz≈*δ* (**a**–**c**) and Ω≈11.9 MHz>8.8 MHz≈*δ* (**d**–**f**) corresponding to the selective and the Λ-regime, respectively. Blue, red and green colours represent the states 
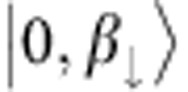
, 
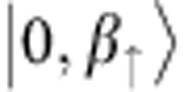
 and 
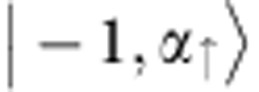
 respectively. (**a**,**d**) Optically detected experiments determining the eigenstate populations at the end of the polarization phase during 30 μs as a function of the MW frequency *ω*. (**b**,**e**) Predicted eigenstate population spectra calculated without free parameters, that is, using calibrated and known couplings, and a hyperfine tensor was taken from ref. 29. In the selective regime (**a**–**c**), the 
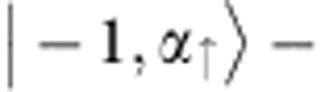
population spectrum exhibits two peaks corresponding to the 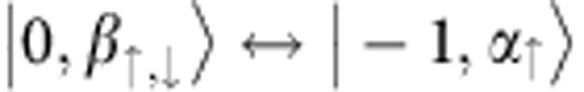 transition frequencies, whereas in the Λ-regime (**d**–**f**) the 
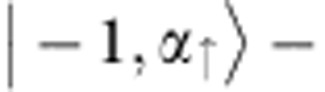
population spectrum has a single peak centred half-way between the 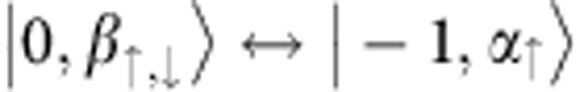 transition frequencies. (**c**,**f**) Derived ^13^C polarization spectra confirming the polarization directions along the 
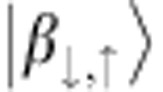
-state axis (**c**) and the 
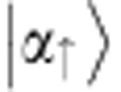
-state axis (**f**) for the selective and Λ-regimes, respectively.

To demonstrate these features, the local ^13^C polarization achieved via this HF-mediated polarization transfer was probed by optically detected magnetic resonance experiments on a single NV centre coupled to a first-shell ^13^C nuclear spin, characterized by a strong HF coupling Δ≈130 MHz (refs 26, 29). ^13^C polarization was measured on a crystal lattice rotated on purpose away from the external magnetic field by arbitrary Azimuthal and polar angles. The electron spin state was initialized to the *m*_s_=0 state by laser light and simultaneously irradiated with MWs for 30 μs. The eigenstate populations were then optically measured by monitoring the fluorecense emited by the NV electronic transitions, as a function of the MW frequency *ω* over a range covering the various transition frequencies (Fig. 2; see Methods, Supplementary Notes 2–4 and Supplementary Figs 5–8 for further details). Two MW powers were used in these experiments, corresponding to the selective and Λ-regimes shown in Fig. 1. For the selective regime, a ^13^C hyperpolarization pointing along the 
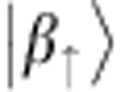
 or 
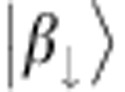
 directions defined by the *m*_s_=0 eigenstate manifold is reached, depending on the MW irradiation frequency. For the Λ-regime, nuclear hyperpolarization is also reached but now aligned along an 
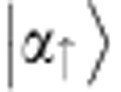
 axis defined by the nuclear eigenstates in the *m*_s_=−1 manifold. The double-peak pattern of the eigenstate population and ^13^C polarization spectra is characteristic of the selective regime. These two ^13^C spin alignment maxima arise when the MW is on resonance with the 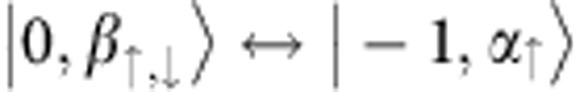 transition frequencies, leading to opposite nuclear polarization directions. By contrast, in the Λ-regime, a single ^13^C spin alignment peak centred between the 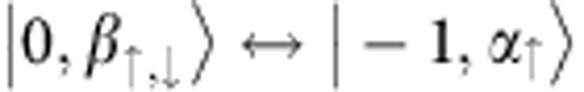 transition frequencies is observed, evidencing that these two transitions are affected by the MW. In such a case the initial populations of the 
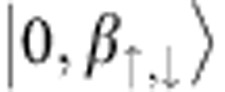
 states are transferred to populate 
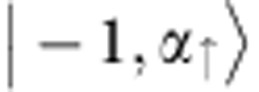
, leading to a nuclear spin alignment defined by the 
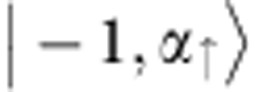
 nuclear quantization axis.

### Bulk hyperpolarization at variable fields and orientations

**Figure 3:**
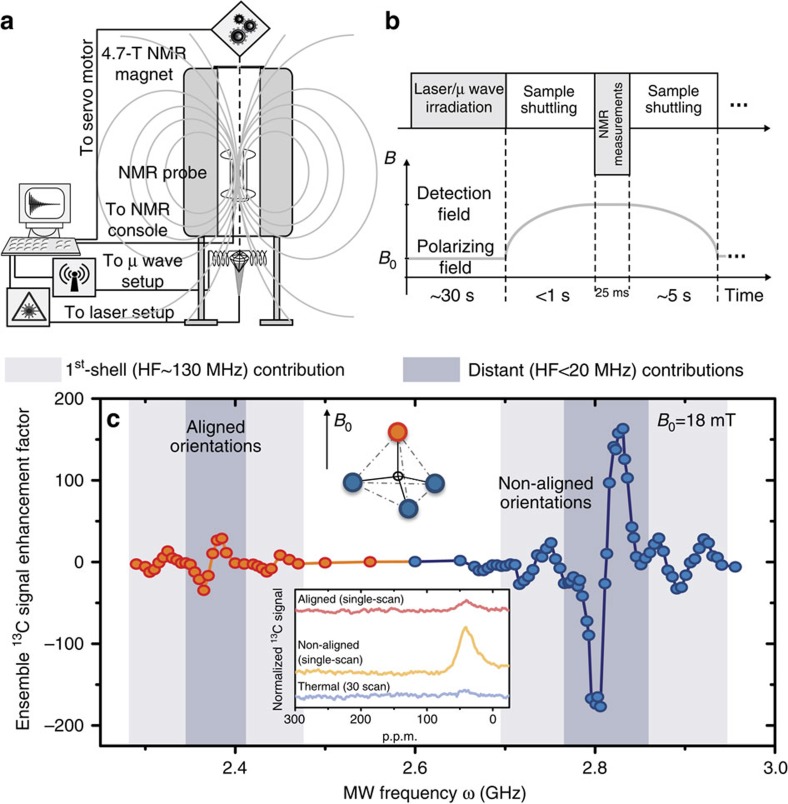
Acquiring ensemble ^13^C polarization spectra for varying NV orientations with respect to **B**_**0**_. (**a**) Opto-NMR set-up and (**b**) detection sequence used in these experiments. During the polarization transfer phase, the entire single-crystal diamond is irradiated with laser light and MW underneath the NMR magnet at a low *B*_0_. The hyperpolarized diamond is then shuttled (in <1 s) into a 4.7-T superconducting magnet to directly detect its macroscopic ^13^C magnetization via a spin-echo sequence. The low *B*_0_ magnetic field is aligned to one of the nitrogen-vacancy-centre orientations (in red), while the other three orientations (in blue) subtend an angle of ≈109° with respect to the field. (**c**) Typical ^13^C polarization enhancement patterns observed by NMR as a function of the MW frequency *ω* with signals normalized with respect to the thermal ^13^C response at 4.7 T (inset). The left part of the plot corresponds the nuclear polarization generated by 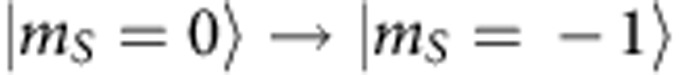 MW transitions for the aligned orientation (red circles), while the right part corresponds to nuclear polarization enhanced via the three non-aligned, equivalent orientations (blue circles). The ≈1:3 intensity ratio reflects the relative abundances of aligned and non-aligned sites in the diamond's tetrahedral structure. In each of the patterns, the central peaks represent bulk nuclear hyperpolarization pumped via ^13^C spins coupled with hyperfine interactions lower than 20 MHz, while the outer peaks originate from first-shell ^13^Cs whose hyperfine splitting is ≈130 MHz (refs 26, 29). The antiphase structure of each of these peaks corresponds to the MW transitions 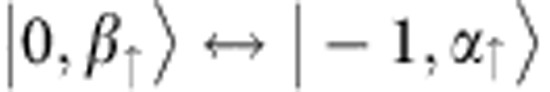 and 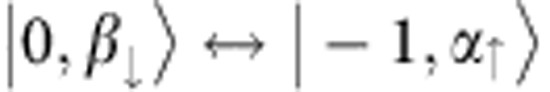 at one side of the central peaks, and to the 
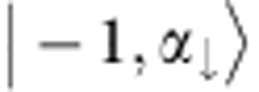
 state at the other side. The inset shows NMR spectra obtained for a thermally polarized sample, and at the maxima of the central peaks for the aligned and non-aligned orientations. p.p.m. refers to parts-per-million of the high-field NMR ^13^C resonance frequency, which in our case is 50.5 MHz.

**Figure 4:**
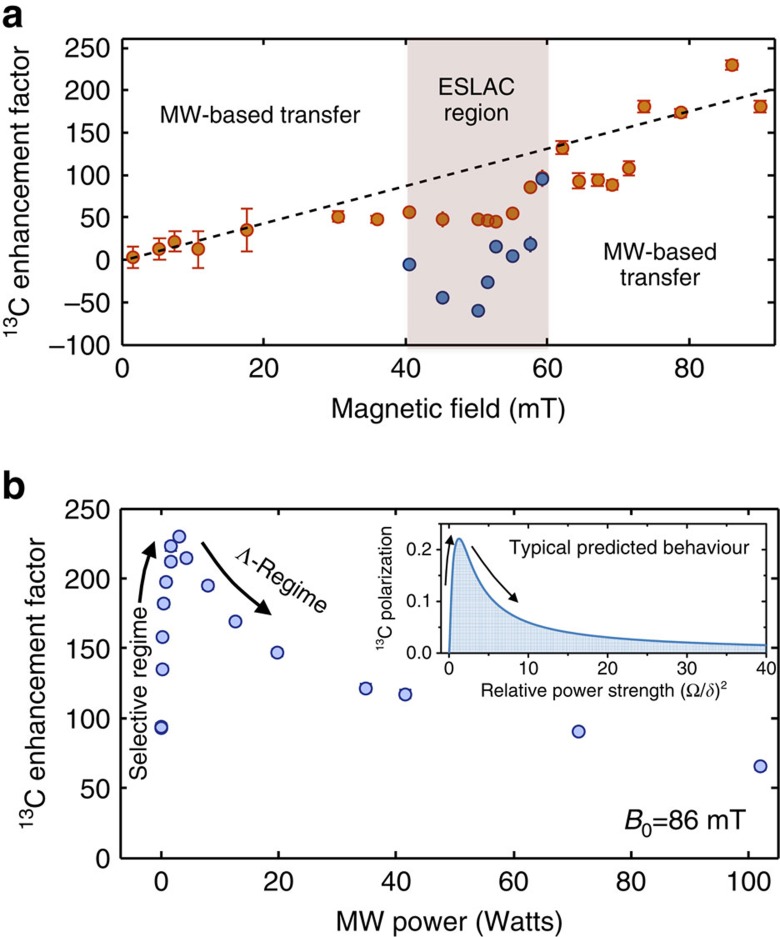
Ensemble ^13^C magnetization for different magnetic field strengths and MW powers. The experimental points of these panels correspond to the maximum signal of the central peak in the aligned orientation. (**a**) ^13^C polarization as a function of the magnetic field strength *B*_0_ (normalized to its thermal 4.7-T counterpart and measured up to our maximum operating field, corresponding to ≈100 mT). The dashed line is a guide to the eye. Within the shaded excited-state level anticrossing (ESLAC) region polarization transfer was observed, deriving from the combined action between a level anticrossing mechanism^12^,^13^,^14^ and the here proposed MW-induced transfer scheme. These two nuclear polarization mechanism proceeded with opposing signs. Within this region, the blue dots show the resulting signal from the combined action of the ESLAC- and MW-induced transfer, while the orange dots show the MW-induced enhancement factor after a phenomenological subtraction of the ^13^C polarization transferred solely by the ESLAC scheme. (**b**) ^13^C hyperpolarization as a function of the MW power at a fixed field of ≈86 mT. The inset shows the typical predicted behaviour by the model, where the ^13^C polarization grows with MW power within the selective regime, but decreases as the Λ-regime is reached. As a qualitative comparison, the inset's numerical simulations consider a single NV centre coupled to a single ^13^C with an hyperfine interaction *A*_*zz*_=5*A*_*zx*_. In this case Δ=80*δ*, the laser-pumping rate=1.5/*δ*, the total polarization time=120/*δ* and the MW frequency is on resonance with the 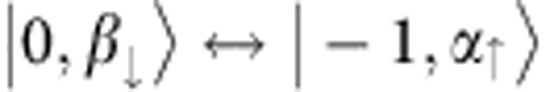 transition. Qualitatively similar behaviours are obtained for different parameters. In both panels, the error bars represent the propagated relative error of the spin signal on the calculated enhancement factors.

The polarized NV centre is embedded in a network of dipole–dipole-interacting nuclear spins, allowing an eventual propagation of the hyperpolarization throughout the nuclear bulk ensemble (Fig. 1a). Achieving an effective ^13^C polarization enhancement throughout an entire diamond could have important practical NMR and MRI consequences as the source of polarization for samples in contact with the diamond, as well as for tracing purposes. Consequently, we investigated whether distant ^13^C spins can benefit from the polarization transfer mechanisms observed in Fig. 2 at a local level. The bulk macroscopic ^13^C magnetization was directly measured by mechanically shuttling the diamond from low fields, where MW and laser fields were applied to accomplish the electron–nuclear polarization transfer, into a 4.7-T magnet enabling bulk ^13^C NMR detection (Fig. 3a,b). Figure 3c presents results arising from a typical experiment, showing the dependence of the ensemble ^13^C magnetization on the MW frequency *ω*. For consistency, measurements were carried out with the polarizing *B*_0_ field aligned along one of the NV centre orientations, meaning that the remaining three orientations of the crystal form an angle of ≈109° away from the field direction. The nuclear polarization spectrum (Fig. 3c) shows two regions of enhancement, corresponding to the aligned and non-aligned orientations of the NV defects. Both regions show similar patterns of multiple positive and negative peaks, corresponding to different HF couplings and MW-induced transitions. Both ^13^C patterns contain major central peaks, and minor outer peaks detuned by ≈±60 MHz from their centres. The central peaks correspond to bulk ^13^C nuclei being hyperpolarized via 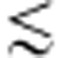
20-MHz HF interactions, that is, via ^13^C spins positioned at or beyond the NV's second shell^27^,^28^. The outer peaks also correspond to bulk ^13^C polarization, but arriving this time via first-shell ^13^Cs whose HF splitting is ≈130 MHz (refs 26, 29). In this instance, the bulk ^13^C magnetization exhibits a sign-flip pattern characteristic of the selective regime, akin to the local ^13^C polarization pattern shown in Fig. 2c, although these are bulk ^13^C resonances detected on the whole diamond, and not single-spin measurements. This demonstrates that ^13^C ensemble hyperpolarization can be derived via either nuclear–nuclear dipole couplings involving distant spins to the electron, or via first-shell ^13^C spins. The latter is a remarkable fact, given that in this case the build-up of bulk polarization needs to overcome the >60-MHz detuning characterizing the first-shell sites acting as polarization transfer bridgehead. The timescales that are then needed to achieve a bulk polarization build-up, ∼30 s, are orders of magnitude longer than the first-shell electron-^13^C polarization transfer time (<ms, see Supplementary Note 4 and Supplementary Fig. 7). This difference in timescales explains how a low-concentration, highly detuned species such as the first-shell ^13^C suffices to polarize the bulk sample examined by the NMR experiments (see Supplementary Note 5 for a rough estimation of the number of polarized ^13^C). Figure 4 further demonstrates the versatility of the method to yield bulk nuclear hyperpolarization, as evidenced by the achievement of enhanced ^13^C NMR signals over a broad range of polarizing magnetic fields and of MW powers. Notice that the nuclear polarization reveals a systematic increase as a function of *B*_0_ (Fig. 4a), as well as a clear optimum for the MW power (Fig. 4b) consistent with a transition from a selective regime in which the polarization grows with MW power to a Λ-regime in which it decreases with the MW power (see inset and Supplementary Note 4). Interference effects between a hyperpolarization driven by an excited-state level anticrossing^13^,^14^,^15^ and the MW-induced transfer scheme introduced here are seen at fields of ∼50 mT. In contrast to the MW-induced transfer, the anticrossing-based technique requires that the electron/nuclear Zeeman energy detuning conditions be smaller than the dominant HF interaction. The relatively narrow range of magnetic field values compatible with this transfer scheme is thus in contrast to the MW-induced transfer processes, where matching requirements are relaxed as the MW frequency can be tuned to an array of electronic–nuclear frequency transitions (for example, Fig. 4a).

## Discussion

A versatile method for hyperpolarizing nuclear spin ensembles at room temperature using NV centres in diamond was proposed and demonstrated. The method relies on the combined action of continuous laser light and MW irradiation, and exploits the asymmetries of the HF interaction imparted by the spin-1 nature of the NV centre. This allows us to transfer spin order from the electronic *m*_s_=0 state to the nuclei, by exploiting the evolution triggered on selectively irradiating 
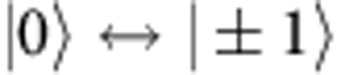
 transitions. Nuclear spin hyperpolarization is thus obtained for a broad range of magnetic field strengths and arbitrary field orientations with respect to the diamond lattice, as well as for a wide range of HF interactions. These principles may facilitate a number of applications. One entails facilitating the use of pre-polarized diamonds in NMR experiments owing to the ease with which NV electrons can be optically pumped by comparison with cryogenic-based high-field counterparts. This easing of the hyperpolarization conditions should also facilitate the use of these samples in multiscan acquisition of the kind desirable in *in vivo* MRI scans. Our scheme provides an approach for optically detected nanoscale NMR/MRI of polarized nuclear spin ensembles that complements methods based on spin noise thermal fluctuations^23^,^32^,^33^,^34^,^35^. The fact that the method works for different orientation and strengths of the magnetic field can be exploited for polarizing diamond powders, leading to an alternative route to NMR measurements that complements the ones based on thermal polarization. Further studies on the basis of frequency sweeps are in progress to evaluate the efficiency of the nuclear spin polarization process in powders at higher fields. Alternatively, approaching a zero magnetic field might be advantageous, since all orientations would then become nearly equivalent and addressable in a simultaneous manner. In addition to potential NMR/MRI uses, this method could be useful for dissipative nuclear-state preparation in ensemble quantum memories, for quantum information processing^36^,^37^,^38^,^39^. Hyperpolarizing nuclear spin ensembles in solid-state systems also provides a tool to study condensed matter and many-body physics phenomena of cold bodies, where quantum effects (for example, quantum phase transitions) are more sharply manifested^40^,^41^,^42^,^43^. The concepts here used might also find applicability in other kinds of cross-polarization scenarios, involving generic transfers between a spin-1 source coupled to other spins.

Besides demonstrating the method's capability at a local level, it was shown that nuclear hyperpolarization can be extended throughout the bulk ensemble by spin-diffusion—even when targeting first-shell nuclear spins strongly shifted by HF couplings. This provides a new tool for understanding the complex non-equilibrium dynamics of many-body systems, where a local polarization can be created in a controlled way and its spreading into the bulk be monitored^44^,^45^,^46^,^47^.

## Methods

### Single-crystal sample

For the single NV detection experiments the sample used was a commercially available, untreated, type IIa, electronic-grade natural abundance ^13^C diamond crystal (dimension: 2 × 2 × 0.3 mm^3^). For the NMR experiments, an isotopically enriched (10% ^13^C) type Ib high-pressure, high-temperature diamond was used. The enriched crystal was grown by the temperature gradient method at a pressure of 6 GPa and a temperature of 1,700 K from Ni-2 wt% Ti solvent using a powdered mixture of ^13^C-enriched and natural abundance graphic carbon. These samples were irradiated at room temperature (2 MeV, 10 h, total fluence 8 × 10^17 ^e cm^−2^) and annealed (2 h, 1,000 °C). Subsequent examination using confocal laser microscopy confirmed that the two most abundant paramagnetic impurities consisted of electrically neutral single substitutional nitrogen atoms (P_1_), and charged nitrogen atoms next to a lattice vacancy forming an optically active colour centre (NV^−^). The concentrations of these impurities were both <5 p.p.m. (part-per-million). In both samples, we estimate a *T*_1_≈5 ms for the NV electron spin in the electronic ground state. Whereas single NV centre experiments on natural abundance ^13^C samples revealed a 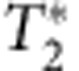
≈2.5 μs (ref. 48), for the ^13^C-enriched crystal we estimate using optically detected magnetic resonance (ODMR) a 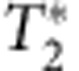
≈12 ns for the NV centre in the ground state at the polarizing fields. For the single NV centre experiments, a 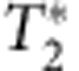
≈20 μs was measured for the nuclear spin. In the ^13^C-enriched diamond crystal, bulk ^13^C *T*_1_ relaxation times ranged from 5 to 20 s depending on the magnetic field strength at the polarizing fields (1–100 mT). At the detection field *B*_0_=4.7 T used in the ^13^C bulk studies, *T*_1_ relaxation was >10 min and 
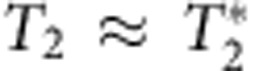
=160±50 μs.

### System Hamiltonian

At room temperature, the NV centre energy-level structure exhibits an electronic triplet as the ground state (^3^*A*_2_)^12^,^13^,^14^. The quantum Hamiltonian of a single NV defect (*S*) and one ^13^C nucleus (*I*) can thus be described as 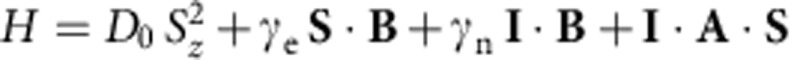. Here *D*_0_=2.87 GHz is the zero-field splitting term, *γ*_e_ and *γ*_n_ the electronic and nuclear gyromagnetic ratios, **B** is the magnetic field vector and **A** a HF tensor that depends on the specific NV and nearby ^13^C spin^49^. For simplicity, we consider first a magnetic field **B** aligned with the axis of the zero-field tensor **D**_**0**_ and the secular approximation 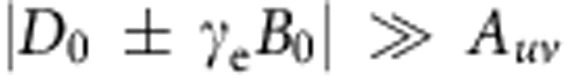. This Hamiltonian simplifies to 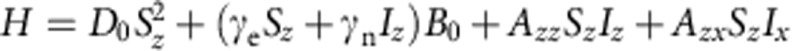, where the axis *x* was chosen such that *A*_*zy*_=0. The feature enabling the magnetization transfers illustrated in Fig. 1 is that in the *m*_s_=0 manifold the eigenstates have a nuclear component 
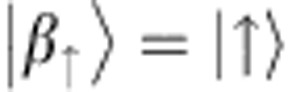
 and 
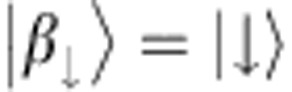
 exhibiting a nuclear Zeeman splitting *δ*=*γ*_n_*B*_0_, whereas in the *m*_s_=−1 manifold the 
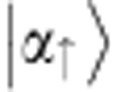
 and 
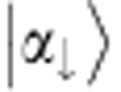
 nuclear components of the eigenstates are quantized on a different axis determined by the HF tensor for weak magnetic fields 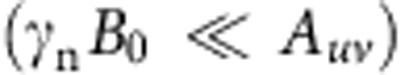. For the 
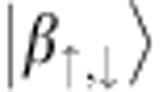
 states the eigenstate splitting *δ* may also depend on non-secular corrections of the HF coupling, which may also lead to an effective tilt of the nuclear spin quantization away from the magnetic field **B** (see Supplementary Figs 1 and 2). By contrast, the 
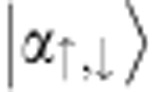
 states are not significantly modified by the non-secular HF terms if 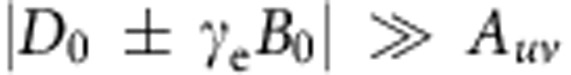, that is, on a condition far from the level anticrossings^13^,^14^,^15^ (we do not consider here the more complicated scenario when the level anticrossing conditions are fulfilled, see Supplementary Fig. 2 for further details). If the magnetic field is not aligned with the **D**_**0**_ tensor, the level structure discussed in Fig. 1 remains even if the definitions of 
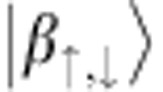
 and 
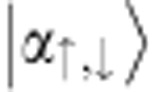
 will change^29^; the crucial characteristic is that they will still have different quantization axes and different eigenstate splittings *δ* and *Δ* (see Supplementary Figs 3 and 4). For more details on the eigenstates, energy splittings and the participation of them on the polarization dynamics please refer to Supplementary Note 1.

### Single NV optical set-up

The single NV detection was conducted on a home-built confocal microscope. Continuous wave green laser light (*λ*=532 nm) was switched by an acousto-optic modulator (Isomet, rise/fall times ≈30 ns) coupled into a single mode optical fibre and focused on the diamond sample by an oil-immersion microscope objective (Olympus, numerical aperture=1.35). The objective was placed on a three-axis piezo stage (Npoint) enabling a scan range of the *x*, *y*, *z* axes of 250 × 250 × 25 μm with a resolution of ∼1 nm. The emerging red fluorescence signal was focused into a single-photon counting module (Perkin-Elmer, dark count rate 100 counts s^−1^). To induce transitions within the ground-state triplet during the polarization and detection sequences, MW fields were produced by two copper wires attached to the diamond (diameter ∼50 μm). During the hyperpolarization process, the laser intensity was reduced to ∼5% of its saturation intensity (*P*_sat_∼1 mW at the objective's back) to avoid light-induced nuclear-state depopulation^12^,^50^ (see Supplementary Note 4 and Supplementary Fig. 8).

### Populations and polarization in single NV centre experiments

The eigenstates' populations were obtained by performing a series of complementary measurements involving selective MW irradiation on eigenstate-specific transition frequencies, followed by an optical readout of the NV centre populations (see Supplementary Note 2 and Supplementary Fig. 5 for further details). With these selective experiments and a linear system of equations, the populations of the three active eigenstates (
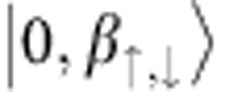
 and 
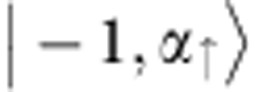
) could be reconstructed. To perform these calculations, the fluorescence levels at the end of the different polarization sequences were measured, and properly normalized by executing the schemes with and without MW irradiation (Supplementary Fig. 6). In the selective regime, the MW Rabi frequency *Ω*=1.4 MHz was lower than the HF splitting with the ^14^N nuclear spin (2.16 MHz)^51^. Therefore, to address the latter's three nuclear spin orientations simultaneously, the MW source was further modulated to create the three corresponding frequencies spaced by 2.16 MHz (see Supplementary Note 3). The wiggles in Fig. 2a–c are consequence of this irradiation mechanism. The magnetic field strength and orientation were optimized to be large enough for allowing selective excitation independent of the host ^14^N nuclear spin (*δ*>4–5 MHz), and small enough to allow mutual excitation (that is, Λ-regime) with reasonable MW power (*δ*<10 MHz). The static and MW magnetic fields and the laser-pumping rate were calibrated from ODMR spectra, and from time-resolved measurements of the Rabi oscillations and the laser-pumping evolution. The values of the static and MW fields are given in the caption to Fig. 2, while the optical pumping rate was 1/3 μs^−1^ and had a pumping efficiency of ≈90%. The curves in Fig. 2b,c,e,f were obtained by solving the spins' quantum evolution based on a quantum master equation involving the total six-level model of a single NV centre coupled to a single ^13^C nuclear spin, as further detailed in Supplementary Note 2. The bare system Hamiltonian used was introduced in the Methods section: System Hamiltonian (for further details see Supplementary Note 1); simulations were performed on the basis of this Hamiltonian in the rotating frame of the MW irradiation, with frequency parameters as discussed in the main text. The pumping process was modelled as a non-Hermitian relaxation process using a Lindblad form reflecting the incoherent transition from the *m*_s_=±1 states to the *m*_s_=0 state, and using the experimentally determined pumping rate 1/3 μs^−1^ as the relaxation rate. The HF tensor of the first-shell ^13^C spin was taken from ref. 29 (see Supplementary equation (16)). For the selective regime, the ^14^N HF splitting was considered phenomenologically as an ensemble superposition of the signal coming from the three frequencies used in the MW irradiation.

### Set-up for the ensemble-detected nuclear spin polarization experiments

In a single-crystal diamond, NV centres can be separated into subensembles corresponding to four magnetically inequivalent orientations. The diamond sample used for the optically pumped NMR measurements had a polished [100] surface orientation. The sample was rotated in *B*_0_ until one of the NV centre orientations was aligned to the magnetic field, while the three remaining subensembles were degenerate at an angle of ∼109° forming the non-aligned orientations. The weak magnetic field in these experiments (*B*_0_=0–100 mT) was provided by the stray field of the superconducting magnet used to perform the bulk high-field NMR measurements, connected by a compensating low-field coil. The laser light was produced by a diode-pumped solid-state laser (Verdi-V10, *λ*=532 nm) coupled into an optic fibre and then focused to either partially or fully illuminate the diamond crystals. To avoid excessive heating during the optical pumping, the sample was immersed into a transparent water-filled flask and vented with pressurized air to keep the temperature constant. For the application of continuous wave MW irradiation, radio- and MW signals from a broadband frequency synthesizer were amplified by up to +30 dB for frequencies *ω*>600 MHz and up to +43 dB for *ω*<600 MHz. These amplified signals were then fed into a Helmholtz coil electronic circuit tuned to 50 Ω.

The bulk ^13^C experiments began with a combined laser and MW irradiation (10–30 s) to optically initialize the electronic spins and induce a transfer of the ensuing hyperpolarization from the electronic to the nuclear spins. The sample was then rapidly (<0.5 s) shuttled from the polarizing field *B*_0_ to the sweet spot of the high-field (4.7 T) NMR magnet, where the nuclear spins were subject to a pulsed detection using an NMR probe with a Helmholtz coil configuration tuned to 50.55 MHz. The observed NMR signal consists of a single optically enhanced ^13^C spectral line resonating at *ca.* 60 p.p.m. with a linewidth between 1 (natural abundance) and 3 kHz (10% enriched crystal). The sample then returned to the polarizing field for a repeated cycle of optical pumping and MW irradiation for the purpose of signal averaging.

## Additional information

**How to cite this article:** Álvarez, G. A. *et al.* Local and bulk ^13^C hyperpolarization innitrogen-vacancy-centred diamonds at variable fields and orientations. *Nat. Commun.* 6:8456 doi: 10.1038/ncomms9456 (2015).

## Supplementary Material

Supplementary Information Supplementary Figures 1-8, Supplementary Notes 1-5 and Supplementary References.
