# Supplementary material for: Local and bulk 13C hyperpolarization in nitrogen-vacancy-centred diamonds at variable fields and orientations
Source: Nat Commun. 2015 Sep 25;6:8456. doi: 10.1038/ncomms9456 (PMC4598721; doi:10.1038/ncomms9456)
Supplement: Supplementary Information — Supplementary Figures 1-8, Supplementary Notes 1-5 and Supplementary References. [file ncomms9456-s1.pdf]

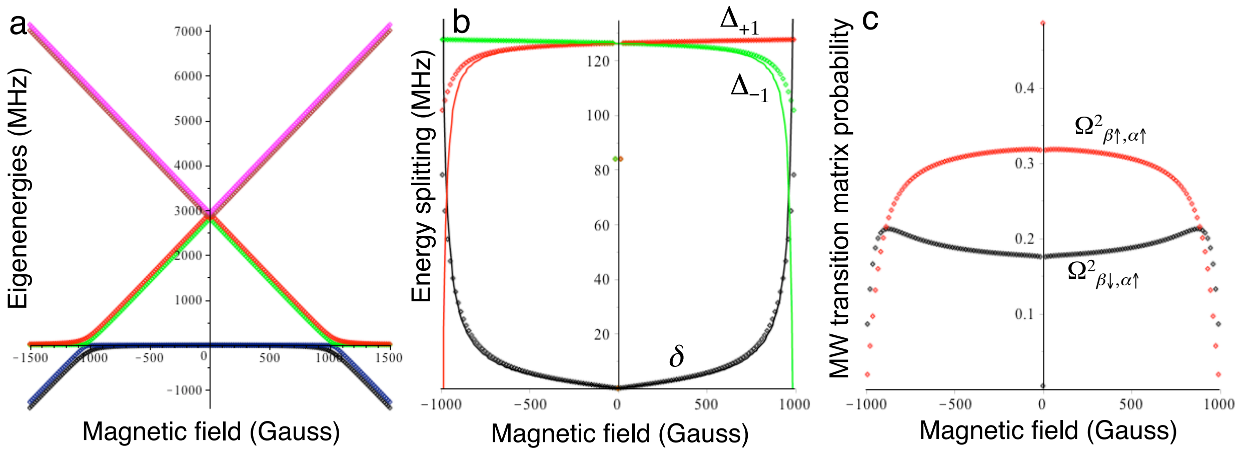

Supplementary Figure 1. **Eigenenergies and microwave transition matrix elements as a function of the magnetic field strength for 1st-shell ^{13}C hyperfine coupled spins.** The magnetic field is assumed aligned with the zero-field tensor main axis. (**a**) Eigenenergies E_i as a function of the magnetic field B_0. At $B_0 = 1000$G there are level anti-crossings, not considered in our scheme. (**b**) Energy splittings δ (black), Δ_{-1} (green) and Δ_{+1} (red) of the corresponding electron spin $m_s = 0, \pm1$ manifolds are shown as a function of the magnetic field strength B_0. The symbols are exact numerical calculations using the hyperfine tensor of Supplementary Eq. (16) and the solid lines are derived from second order perturbation theory (Supplementary Eqs. (8) and (9)), which were determined by 2nd order perturbation theory. (**c**) Microwave transition matrix elements $\Omega^2_{\beta_{\uparrow,\downarrow},\alpha_\uparrow} = |\langle 0, \beta_{\uparrow,\downarrow}| \, S_x \, |-1, \alpha_\uparrow\rangle|^2$, found to be in general both non-null. Similar curves are obtained for $|\langle 0, \beta_{\uparrow,\downarrow}| \, S_x \, |-1, \alpha_\downarrow\rangle|^2$, and for $|\langle 0, \beta_{\uparrow,\downarrow}| \, S_x \, |+1, \alpha_{\uparrow,\downarrow}\rangle|^2$.

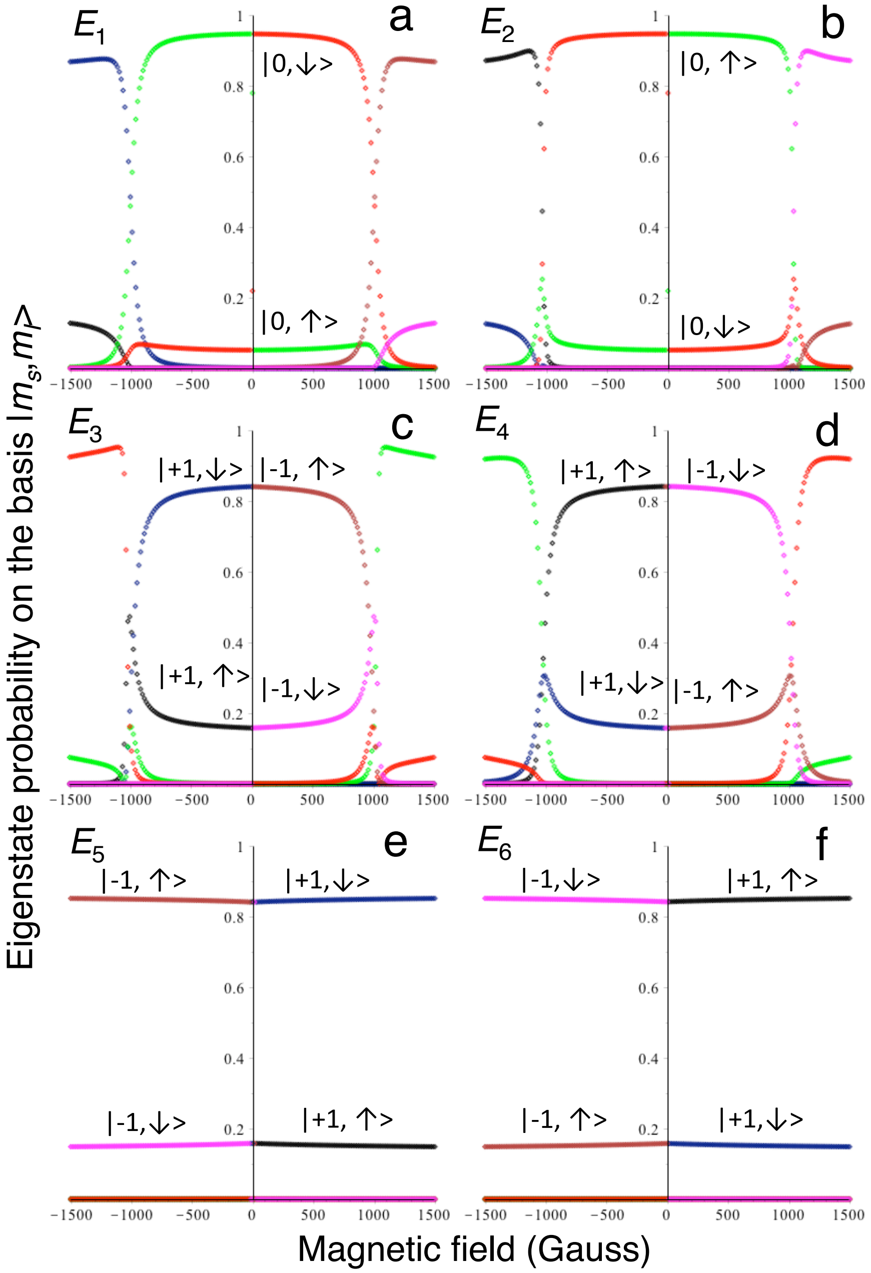

Supplementary Figure 2. **Eigenstates description in the $|m_S, m_I\rangle$ basis as a function of the magnetic field strength for the 1st-shell ^{13}C hyperfine interaction.** The magnetic field is aligned to the zero-field tensor axis. Each panel shows the probabilities $|\langle m_S, m_I|E_i\rangle|^2$ of the different eigenstates $|E_i\rangle$ of the system, where i is an eigenenergy index such that the eigenenergies follow $E_i \leq E_{i+1}$ according to the panel label. The color-coded symbols represents the different states $|m_S, m_I\rangle$ as red ($|0, \downarrow\rangle$), green ($|0, \uparrow\rangle$), magenta ($|-1, \downarrow\rangle$), brown ($|-1, \uparrow\rangle$), blue ($|+1, \downarrow\rangle$) and black ($|+1, \uparrow\rangle$).

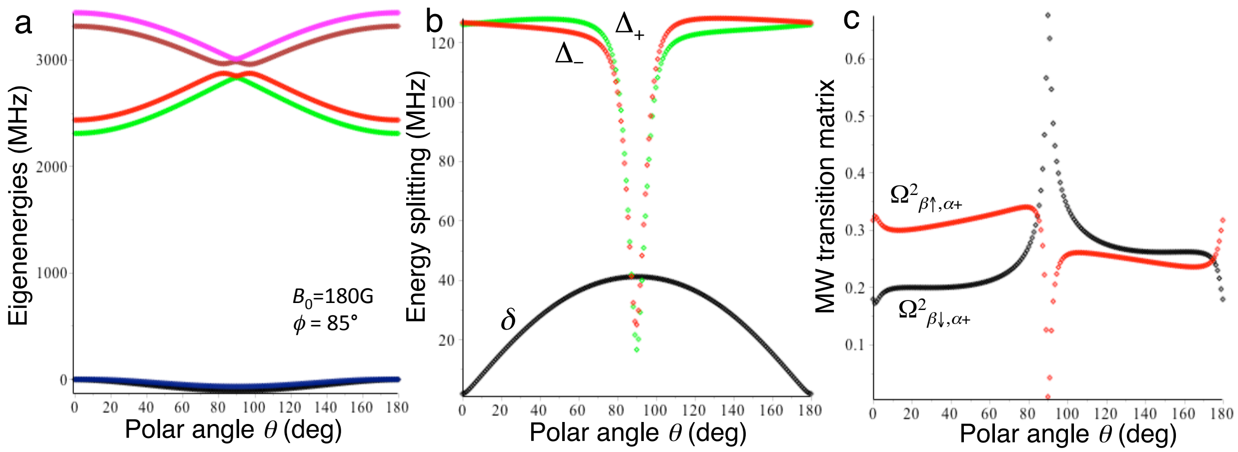

Supplementary Figure 3. Eigenenergies and microwave transition matrix elements as a function of the polar angle θ between the magnetic field and the zero-field main axis for the 1st-shell ^{13}C hyperfine coupled nuclei. The magnetic field strength is $B_0 = 180$G and the azimuthal angle $\phi = 85°$. (**a**) Eigenenergies E_i as a function of the polar angle θ. (**b**) Energy splittings $\delta = E_2 - E_1$ (black), $\Delta_- = E_4 - E_3$ (red) and $\Delta_+ = E_6 - E_5$ (green) between two consecutive eigenenergies in (**a**). δ can be approximated by Supplementary Eq. (15). (**c**) Microwave transition matrix elements $\Omega^2_{\beta_{\uparrow,\downarrow},\alpha_-} = |\langle 0, \beta_{\uparrow,\downarrow}| S_x |-, \alpha_+\rangle|^2$ are shown to be in general both non-null. The eigenstate $|-, \alpha_-\rangle$ is the one corresponding to the eigenenergy given by the green curve. Similar curves are obtained for $|\langle 0, \beta_{\uparrow,\downarrow}| S_x |-, \alpha_-\rangle|^2$, and for $|\langle 0, \beta_{\uparrow,\downarrow}| S_x |+, \alpha_{-,+}\rangle|^2$.

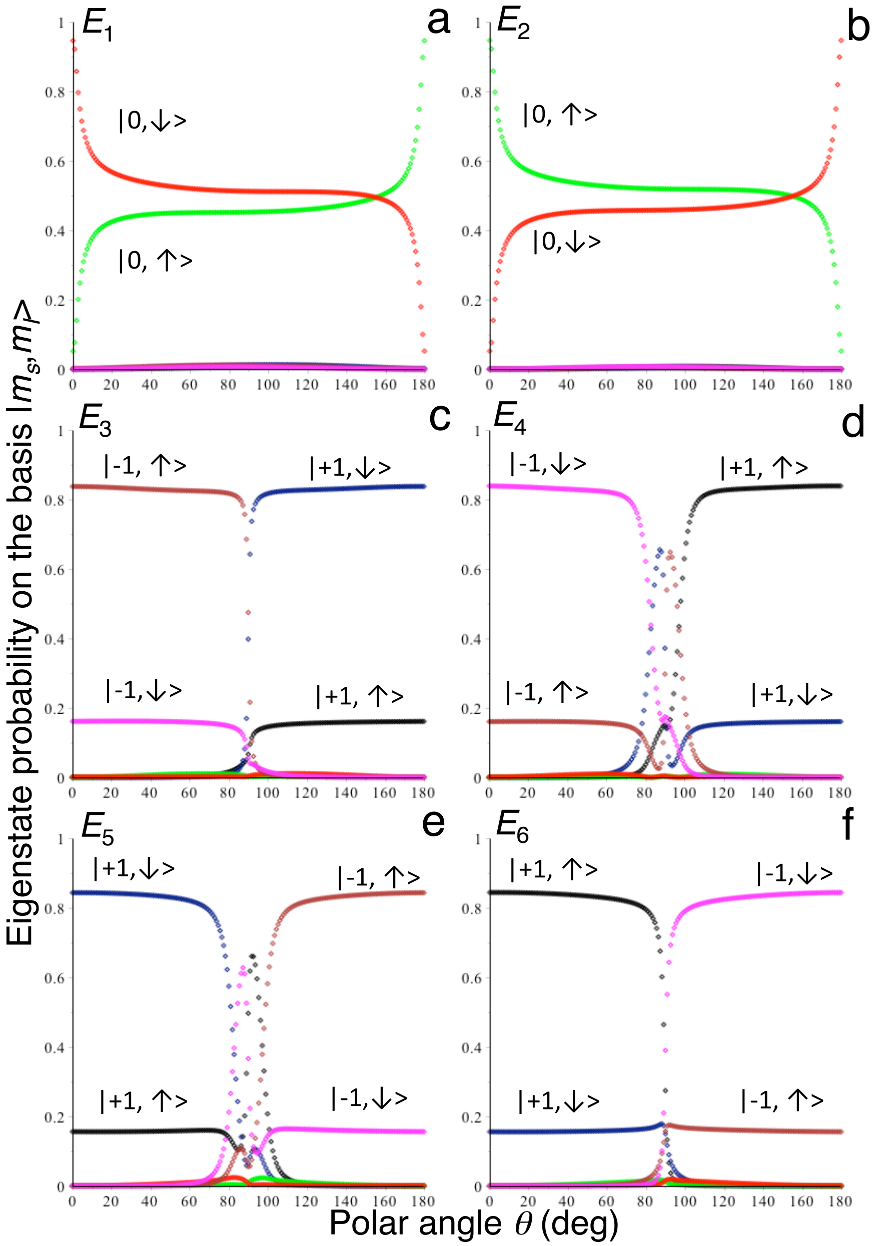

Supplementary Figure 4. **Eigenstates composition in the basis $|m_S, m_I\rangle$ as a function of the polar angle of magnetic field with the zero-field axis for the 1st-shell 13C hyperfine interaction.** The magnetic field strength is $B_0 = 180$G and the azimuthal angle $\phi = 85°$. Each panel shows the probabilities $|\langle m_S, m_I|E_i\rangle|^2$ of the different eigenstates $|E_i\rangle$ of the system, where i is the eigenenergy index such that the eigenenergies are ordered following the rule $E_i \leq E_{i+1}$. The color-coded symbols represents the different states $|m_S, m_I\rangle$ as red ($|0,\downarrow\rangle$), green ($|0,\uparrow\rangle$), magenta ($|-1,\downarrow\rangle$), brown ($|-1,\uparrow\rangle$), blue ($|+1,\downarrow\rangle$) and black ($|+1,\uparrow\rangle$).

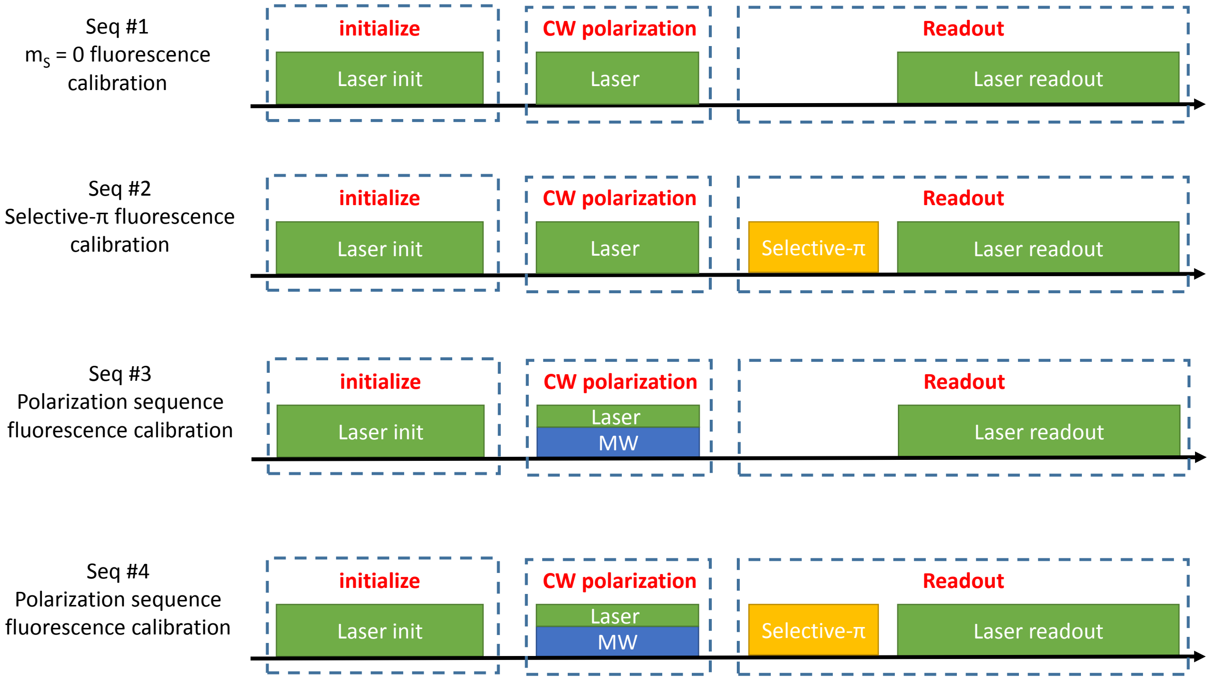

Supplementary Figure 5. Optical detection scheme for quantifying the nuclear spin state populations. The detection sequences are comprised of three stages: initialization, nuclear polarization, and readout. The initialization stage is identical in all sequences, and its main purpose is to completely depolarize the nuclear spin due to the strong optical pumping [1, 2]. The nuclear polarization stage includes a laser pulse and, optionally, a simultaneous microwave irradiation pulse. The sequences lacking microwave excitation are used for fluorescence calibration, and do not generate nuclear polarization. The readout sub-sequence always comprises of a laser readout pulse, and may be preceded by a selective π-pulse, driving one of the $|0, \beta_{\uparrow,\downarrow}\rangle \leftrightarrow |-1, \alpha_{\uparrow}\rangle$ transitions. In our experiments we have chosen the $|0, \beta_{\uparrow}\rangle \leftrightarrow |-1, \alpha_{\uparrow}\rangle$ transition.

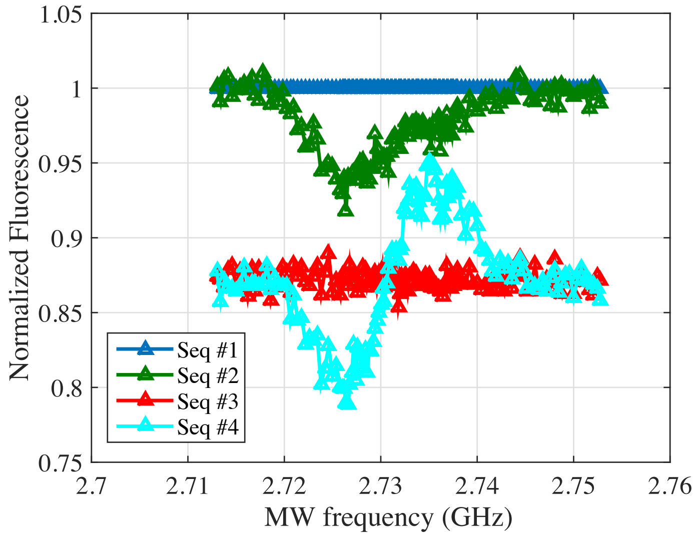

Supplementary Figure 6. Raw optically detected magnetic resonance fluorescence data. It is plotted as a function of microwave frequency, with all outcomes normalized to the fluorescence level of Seq #1.

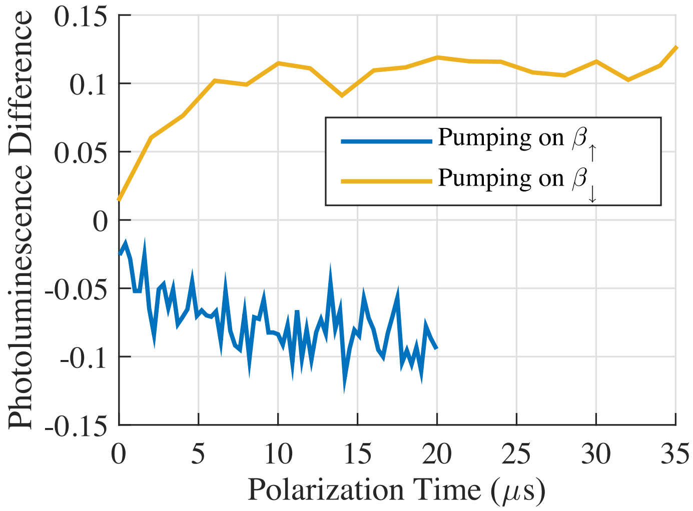

Supplementary Figure 7. Nuclear polarization dynamics. The y-axis is a difference of detection sequence #4 and #2. The microwave frequency of the π-pulse of the detection phase was fixed at the $|0, \beta_\downarrow\rangle \leftrightarrow |0, \alpha_\uparrow\rangle$ transition, while the frequency at the polarization phase was changed with respect to the polarization transition $|0, \beta_\downarrow\rangle \leftrightarrow |0, \alpha_\uparrow\rangle$ (orange) and the $|0, \beta_\uparrow\rangle \leftrightarrow |0, \alpha_\uparrow\rangle$ (blue).

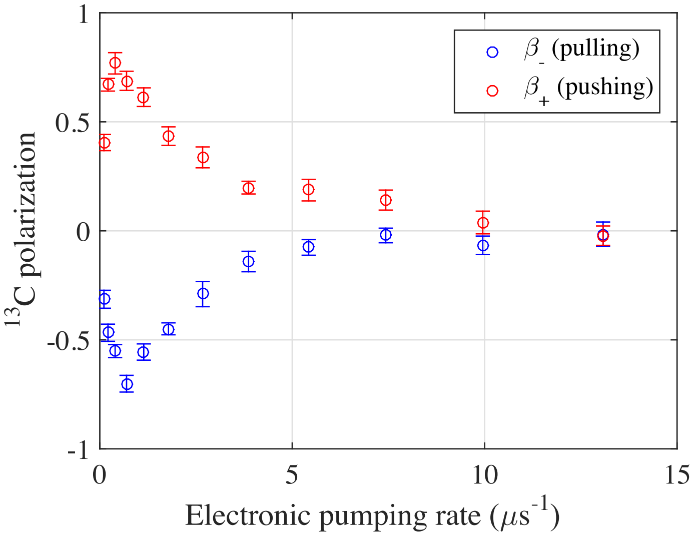

Supplementary Figure 8. **Nuclear spin polarization versus electronic pumping rate.** The blue (red) circle represents a microwave frequency resonant with the $|0, \beta_{\uparrow}\rangle \leftrightarrow |0, \alpha_{\uparrow}\rangle$, ($|0, \beta_{\downarrow}\rangle \leftrightarrow |0, \alpha_{\uparrow}\rangle$) transition. The error bars are the standard deviation of the averaged signal.

Supplementary Note 1

A. System Hamiltonian

Here we give a short summary on the quantum mechanical details of the interaction between a single nitrogen-vacancy (NV) center (S) and a single ^{13}C nuclear spin (I), which is needed for deriving the energy level structure of the coupled system and the microwave (MW) driven polarization scheme. At room temperature the NV-center energy level structure exhibits an electronic triplet as the ground state ($^3A_2$) [1, 3, 4]. The quantum Hamiltonian can thus be described as

$$H = D_0\, S_z^2 + \gamma_{\mathrm{e}}\, \mathbf{S}\cdot\mathbf{B} + \gamma_{\mathrm{n}}\, \mathbf{I}\cdot\mathbf{B} + \mathbf{I}\cdot\mathbf{A}\cdot\mathbf{S}. \tag{1}$$

Here $D_0 = 2.87$ GHz is the zero-field splitting term, γ_{e} and γ_{n} the electronic and nuclear gyromagnetic ratios, $\mathbf{B}$ is the magnetic field vector, and $\mathbf{A}$ a hyperfine (HF) tensor that depends on the specific NV and nearby ^{13}C spin. The important feature to generate the asymmetries of the eigenstates in the different electron spin manifolds is the fact that the electron is a spin 1. Therefore as the hyperfine interaction depends on the quantum magnetic number m_S, this naturally provides an asymmetry between the $m_S = 0$ and $m_S = \pm 1$ subspaces.

B. Hyperfine interaction

The HF coupling between the two spins given in Supplementary Eq. (1), is composed of two terms - a contact term and a dipolar term [5]

$$H_{\mathrm{HF}} = -\mu_{\mathrm{e}}\mu_{\mathrm{n}} \frac{8\pi \left|\psi_{\mathrm{e}}\left(\mathbf{r}_i\right)\right|^2}{3} \mathbf{S}\cdot\mathbf{I}^i + \left\langle \frac{\mu_{\mathrm{e}}\mu_{\mathrm{n}}}{r_i^3} \left(\mathbf{S}\cdot\mathbf{I}^i - 3\left(\mathbf{n}_i\cdot\mathbf{S}\right)\left(\mathbf{n}_i\cdot\mathbf{I}^i\right)\right) \right\rangle, \tag{2}$$

where μ_{e} and μ_{n} are the NV center and the ^{13}C nuclear magnetic moment respectively, $\psi_{\mathrm{e}}\left(\mathbf{r}_i\right)$ is the electronic wavefunction at the ^{13}C position $\mathbf{r}_i$, $\mathbf{n}_i$ is the unit vector connecting the electronic wavefunction and the ^{13}C position. $\langle\rangle$ denotes an average over the electronic wavefunction $\psi_{\mathrm{e}}\left(\mathbf{r}_i\right)$. The hyperfine coupling can thus be represented by a 3×3 symmetric tensor A_{ij}, thus expressing the hyperfine Hamiltonian term as

$$H_{\mathrm{HF}} = \mathbf{S}\cdot\mathbf{A}\cdot\mathbf{I}. \tag{3}$$

C. Eigenenergies and eigenstates for an aligned magnetic field under the secular approximation

For simplicity, we consider first a magnetic field $\mathbf{B}$ aligned with the axis of the zero-field tensor $\mathbf{D_0}$ and a secular approximation for the hyperfine interaction with respect to $D_0\, S_z^2 + \gamma_{\mathrm{e}}\, S_z B_0$, i.e. a scenario where $|D_0 \pm \gamma_{\mathrm{e}} B_0| \gg A_{uv}$. The Hamiltonian is then

$$H = D_0 S_z^2 + \left(\gamma_{\mathrm{e}} S_z + \gamma_{\mathrm{n}} I_z\right) B_0 + S_z \left(A_{zz} I_z + A_{zx} I_x\right), \tag{4}$$

where the axis x was chosen such that $A_{zy} = 0$ without lack of generality. The feature enabling the magnetization transfers illustrated in Fig. 1, is that in the $m_s = 0$ manifold the eigenstates have a nuclear component $|\beta_\uparrow\rangle = |\uparrow\rangle$ and $|\beta_\downarrow\rangle = |\downarrow\rangle$ exhibiting a nuclear Zeeman splitting $\delta = \gamma_{\mathrm{n}} B_0$; whereas in the $m_s = -1$ manifold, the $|\alpha_\uparrow\rangle$ and $|\alpha_\downarrow\rangle$ nuclear components of the eigenstates are quantized on a different axis determined by the hyperfine tensor. If $A_{zz} \gg \gamma_{\mathrm{n}} B_0$, the latter can be approximated as

$$|\alpha_\uparrow\rangle \approx \cos(\eta/2)\,|\uparrow\rangle + \sin(\eta/2)\,|\downarrow\rangle\,, \tag{5}$$

$$|\alpha_\downarrow\rangle \approx \sin(\eta/2)\,|\uparrow\rangle - \cos(\eta/2)\,|\downarrow\rangle\,, \tag{6}$$

where $\tan\eta = A_{zx}/A_{zz}$, and the eigenenergies of these states are split by $\Delta \approx \sqrt{A_{zz}^2 + A_{zx}^2} \gg \delta$. If $\sqrt{A_{zx}^2 + A_{zz}^2} \lesssim \gamma_{\mathrm{n}} B_0$ then A_{zz} has to be replaced by $A_{zz} - \gamma_{\mathrm{n}} B_0$ (or $A_{zz} + \gamma_{\mathrm{n}} B_0$ in the $m_s = +1$ manifold), but the overall physics remains the same. The elements of the MW transition matrix are given by $\Omega_{\beta_{\uparrow,\downarrow},\alpha_{\uparrow,\downarrow}} = \langle 0, \beta_{\uparrow,\downarrow}|\, S_x\, |{-1}, \alpha_{\uparrow,\downarrow}\rangle = 1/\sqrt{2}\,\langle \beta_{\uparrow,\downarrow}|\alpha_{\uparrow,\downarrow}\rangle$; these are in general all non-null, enabling the application of the proposed polarization transfer scheme. In the Λ-regime referred to in the main text, we opted to describe the system on a basis that is suited to the MW selection rules. The main text thus described this regime ($\delta \lesssim \Omega \ll \Delta$) in a basis set $\{|0, \alpha_\uparrow\rangle\,, |0, \alpha_\downarrow\rangle\,, |{-1}, \alpha_\uparrow\rangle\}$; where $|0, \alpha_\downarrow\rangle$ is a dark state for the MW in the sense that $\langle 0, \alpha_\downarrow|\, S_x\, |{-1}, \alpha_\uparrow\rangle = 0$, and $|0, \alpha_\uparrow\rangle$ is a bright state $\langle 0, \alpha_\uparrow|\, S_x\, |{-1}, \alpha_\uparrow\rangle = 1/\sqrt{2}$ that is addressed by the MW. Due to the different HF properties associated with $m_s = 0$ and $m_s = -1$, $|{-1}, \alpha_\uparrow\rangle$ is an eigenstate but $|0, \alpha_\uparrow\rangle$ and $|0, \alpha_\downarrow\rangle$ are not, and therefore oscillate around the magnetic field at the nuclear Larmor frequency $\delta = \gamma_{\mathrm{n}} B_0$.

D. Non-secular effects on eigenenergies and eigenstates for an aligned magnetic field

The previous paragraph assumed that in the $m_s = 0$ manifold, the splitting δ of the eigenstates $|\beta_{\uparrow,\downarrow}\rangle$ was dominated by the nuclear Zeeman term. If B_0 is very small, it may also be relevant to consider the non-secular corrections of the HF coupling, which may lead to an effective tilt of the nuclear spin quantization away from the magnetic field $\mathbf{B}$. By contrast, the $|\alpha_{\uparrow,\downarrow}\rangle$ states are not significantly modified by these non-secular HF terms as long as $|D_0 \pm \gamma_e B_0| \gg A_{uv}$, a condition we will presume fulfilled. To obtain analytical expressions for the energy splittings in this non-secular dominated case, we use second order perturbation theory for evaluating the relevant terms of the HF coupling

$$H_{\mathrm{HF}}^{\mathrm{ns}} = A_{xx} S_x I_x + A_{yy} S_y I_y + A_{xz} S_x I_z. \tag{7}$$

With this we obtain

$$\delta \approx \gamma_{\mathrm{n}} B_0 + \sum_{m_s,u} \frac{|\langle m_s, \alpha_u| H_{\mathrm{HF}}^{\mathrm{ns}} |0, \beta_\uparrow\rangle|^2}{E_{0,\beta_\uparrow} - E_{m_s,\alpha_u}} - \sum_{m_s,u} \frac{|\langle m_s, \alpha_u| H_{\mathrm{HF}}^{\mathrm{ns}} |0, \beta_\downarrow\rangle|^2}{E_{0,\beta_\downarrow} - E_{m_s,\alpha_u}}, \tag{8}$$

$$\Delta_{m_s} \approx \sqrt{(m_s A_{zz} + \gamma_{\mathrm{n}} B_0)^2 + A_{zx}^2} + \sum_{u} \frac{|\langle 0, \beta_u| H_{\mathrm{HF}}^{\mathrm{ns}} |m_s, \alpha_\downarrow\rangle|^2}{E_{m_s,\alpha_\uparrow} - E_{0,\beta_u}} - \sum_{u} \frac{|\langle 0, \beta_u| H_{\mathrm{HF}}^{\mathrm{ns}} |m_s, \alpha_\uparrow\rangle|^2}{E_{m_s,\alpha_\downarrow} - E_{0,\beta_u}}, \tag{9}$$

where $|0, \beta_u\rangle$ and $|m_s, \alpha_u\rangle$ are the eigenstates of the secular Hamiltonian (4) and E_i are the corresponding eigenvalues

$$E_{0,\beta_\downarrow} = -\frac{\gamma_{\mathrm{n}} B_0}{2}, \tag{10}$$

$$E_{0,\beta_\uparrow} = \frac{\gamma_{\mathrm{n}} B_0}{2}, \tag{11}$$

$$E_{m_s,\alpha_u} = D_0 + m_s \gamma_{\mathrm{e}} B_0 + u\sqrt{(m_s A_{zz} + \gamma_{\mathrm{n}} B_0)^2 + A_{zx}^2}, \tag{12}$$

with $u = \pm 1/2$ for $\uparrow$ and $\downarrow$ respectively. The 2nd order corrections are more significant within the $m_s = 0$ subspace. In order to provide an order of magnitude for this correction, we consider the case where $A_{xz} = A_{zx} = A_{xx} = A_{yy} = A_{zz}$. A Taylor expansion to first order of the magnetic field strength gives for the effective Larmor frequency

$$\delta \approx \gamma_{\mathrm{n}} B_0 + \frac{A_{zz}^2}{D_0^2} \gamma_{\mathrm{e}} B_0, \tag{13}$$

where for the 1st-shell ^{13}C discussed in the main text, the non-secular contribution overcomes the secular part $\gamma_{\rm n} B_0$ by a factor of $\frac{A_{zz}^2}{D_0^2}\frac{\gamma_{\rm e}}{\gamma_{\rm n}} \sim 5$ for weak magnetic fields $B_0 \ll D_0/\gamma_{\rm e} \approx 100$ mT.

E. Non-secular effects on the eigenenergies and eigenstates for nonaligned magnetic fields

So far we considered the magnetic field to be aligned with the $\mathbf{D_0}$ tensor. If this ceases to be the case, the level structure discussed in Fig. 1 remains even if the definitions of $|\beta_{\uparrow,\downarrow}\rangle$ and $|\alpha_{\uparrow,\downarrow}\rangle$ will change. The crucial characteristic to point out, is that these $|\alpha_{\uparrow,\downarrow}\rangle$ and $|\beta_{\uparrow,\downarrow}\rangle$ will still have different quantization axes, and different eigenstate splittings δ and Δ. For weak magnetic fields ($\gamma_{\rm e} B_0 \ll D_0$), the secular approximation of Supplementary Eq. (4) needs to be rewritten as

$$H = D_0 S_z^2 + \gamma_{\rm e} B_z S_z + \gamma_{\rm n} \mathbf{B}\cdot\mathbf{I} + A_{zz} S_z I_z + A_{zx} S_z I_x. \tag{14}$$

The secular eigenstates on the $m_s = 0$ manifold will now be defined by the magnetic field direction, i.e. $|\beta_\uparrow\rangle = \cos\frac{\theta}{2}|\uparrow\rangle + e^{\mathrm{i}\phi}\sin\frac{\theta}{2}|\downarrow\rangle$ and $|\beta_\downarrow\rangle = \sin\frac{\theta}{2}|\uparrow\rangle - e^{\mathrm{i}\phi}\cos\frac{\theta}{2}|\downarrow\rangle$, where θ and ϕ are the polar and azimuthal angles subtended by the magnetic field in the principal axis system of the zero-field tensor. A 2nd order perturbation theory analysis was considered in Ref. [6], for these misalignment conditions. For weak magnetic fields $\gamma_{\rm e} B_0 \ll D_0$, one can consider the quantization axis of the zero-field tensor to describe the system states. In this frame, the eigenstates of the $m_s = \pm 1$ manifold are not significantly modified from the ones obtained with the secular approximation. However, the non-secular contribution can turn eventually dominant within the $m_s = 0$ subspace. Therefore, besides the type of corrections considered in Supplementary Eqs. (8) and (9), one now need to consider the non-secular effects due to a non-aligned magnetic field. The quantization axis of the $|\beta_{\uparrow,\downarrow}\rangle$ will then strongly depend on the magnetic field orientation; the effective Larmor splitting between these states was approximated in Ref. [6] as

$$\delta \approx \frac{2\gamma_{\rm e} B_0 \sin\theta}{D_0}\left(\sqrt{A_{xx}^2 + A_{zx}^2}\cos^2\phi + A_{yy}\sin^2\phi\right). \tag{15}$$

Notice that this approximation does not account for the corrections derived in (8), that can be important when the angle θ is small.

F. Numerical calculations of the eigenstates and eigenvalues for the first-shell hyperfine

The components of the hyperfine tensor are dependent on the relative position between the NV center and the ^{13}C nuclear spin. The strongest coupling between the spins occurs for the nearest neighbors - when the ^{13}C is found one lattice site from the vacancy of the NV center. The hyperfine tensor for these first-shell coupling was determined in Ref. [6]

$$\mathbf{A} = \begin{pmatrix} 166.9 & 0 & -90.3 \\ 0 & 122.9 & 0 \\ -90.3 & 0 & 90 \end{pmatrix}, \tag{16}$$

where all values are in MHz. Given this first-shell ^{13}C HF interaction, we consider a numerical analysis of the ensuing eigenvectors and eigenenergies. To this effect we consider first the case where the magnetic field is aligned with the zero-field axis. Supplementary Figure 1a shows the resulting eigenenergies as a function of the magnetic field strength B_0; notice the level-anti-crossing happening at $B_0 = 1000$G. Supplementary Figure 1b shows the energy splittings in the $m_s = 0, \pm1$ manifolds - δ, Δ_{-1} and Δ_{+1} respectively - as a function of the magnetic field strength B_0. The symbols are exact numerical calculations and the solid lines are the approximation of Supplementary Eqs. (8) and (9) based on 2nd order perturbation theory. The polarization in this study is based on the fact that the MW transition matrix $\Omega_{\beta_{\uparrow,\downarrow},\alpha_{\uparrow,\downarrow}} = \langle 0, \beta_{\uparrow,\downarrow}| S_x |-1, \alpha_{\uparrow,\downarrow}\rangle$ elements between eigenstates are all not null; this is shown in Supplementary Fig. 1c for the case $\Omega_{\beta_{\uparrow,\downarrow},\alpha_{\uparrow}} = \langle 0, \beta_{\uparrow,\downarrow}| S_x |-1, \alpha_{\uparrow}\rangle$. Similar curves are obtained for $|\langle 0, \beta_{\uparrow,\downarrow}| S_x |-1, \alpha_{\downarrow}\rangle|^2$, and for $|\langle 0, \beta_{\uparrow,\downarrow}| S_x |+1, \alpha_{\uparrow,\downarrow}\rangle|^2$. To better appreciate the difference between the eigenstates of the different manifolds, Supplementary Fig. 2 shows the probabilities $|\langle m_S, m_I|E_i\rangle|^2$ for the different eigenstates $|E_i\rangle$ in the system, where i is the eigenenergy index ordered such that $E_i \leq E_{i+1}$. For weak magnetic fields $\gamma_e B_0 \ll D_0$ the eigenstates are well separated and described by the quantum number m_S; i.e., they are given by $|0, \beta_{\uparrow,\downarrow}\rangle$ and $|m_S, \alpha_{\uparrow,\downarrow}\rangle$. The $|\alpha_{\uparrow,\downarrow}\rangle$ states are well approximated by Supplementary Eqs. (5) and (6), and are almost independent of the magnetic field strength far way from the level anti-crossings. For the $|\beta_{\uparrow,\downarrow}\rangle$ it can be seen that non-secular terms of the HF change the quantization axis with respect to the one of the Zeeman field for this 1st-shell HF strength, but still they are almost independent of the magnetic field strength far away from the level

anti-crossings. If the HF strength is reduced by one order of magnitude, mimicking the 2nd shell HF interaction, the non-secular effects on the eigenstates become negligible and thus $|\beta_\uparrow\rangle \approx |\uparrow\rangle$ and $|\beta_\downarrow\rangle \approx |\downarrow\rangle$. The same applies for more distant shells.

We now consider the 1st-shell behavior given a misalignment of the magnetic field with respect to the zero-field tensor axis, by a polar angle θ and azimuthal angle ϕ. Supplementary Figure 3 shows the eigenenergies (panel a) and the energy splitting on the different manifolds (panel b) as a function of θ for an arbitrarily chosen $\phi = 85°$ and a magnetic field strength of 180G (the single NV center experiments were done at $\theta = 42°$ and $\phi = 85°$). We define here the manifold of the electron spin state as $+$ and $-$ according to the order of the eigenenergies, because the relevant electron state $m_S = \pm 1$ changes at $\theta = 90°$. Supplementary Figure 3c shows the MW transition matrix elements between the two lowest eigenstates $|0, \beta_{\uparrow,\downarrow}\rangle$ and $|-, \alpha_+\rangle$ (the eigenstate of the green eigenenergy in Supplementary Fig. 3a) evidencing that they are in general both non-null due to the asymmetries of the nuclear components of the eigenstates. Similar curves are obtained for $|\langle 0, \beta_{\uparrow,\downarrow}| S_x |-, \alpha_-\rangle|^2$, and for $|\langle 0, \beta_{\uparrow,\downarrow}| S_x |+, \alpha_{-,+}\rangle|^2$. This is fulfilled in general, also for other azimuthal angles ϕ.

Finally, to identify the relevant electron spin state for the different orientation, Supplementary Fig. 4 shows the probabilities $|\langle m_S, m_I|E_i\rangle|^2$ of the system's different eigenstates $|E_i\rangle$ under these non-aligned conditions. It can be clearly seen that in the $m_S = \pm 1$ manifolds the eigenstates are still mainly determined by Supplementary Eqs. (5) and (6) with the exception of $\theta \approx 90°$. However, the quantization axis of the eigenstates in the $m_S = 0$ manifold strongly depends on the orientation of the magnetic field.

Supplementary Note 2

Optical detection scheme for the nuclear spin state. In our experiments with single NV centers the detection of the nuclear spin state was based on a combination of several sequences, summarized schematically in Supplementary Fig. 5. These sequences aim at reconstructing the populations of the three active eigenstates, namely $|0, \beta_{\uparrow,\downarrow}\rangle$ and $|-1, \alpha_{\uparrow}\rangle$, at the end of the polarization sequence. This is done by measuring the NV center's fluorescence level at the end of the polarization stage, in the presence and absence of MW irradiation. A common feature of the polarization readout sequences is that they are applied with or without a selective MW π-pulse, which exchanges the population of one of the $|0, \beta_{\uparrow,\downarrow}\rangle$ states with that of $|-1, \alpha_{\uparrow}\rangle$. Together, all four sequences enable us to determine the populations of the $|0, \beta_{\uparrow,\downarrow}\rangle$, $|-1, \alpha_{\uparrow}\rangle$ states, assuming that the other three states, namely $|+1, \alpha_{\uparrow,\downarrow}\rangle$ and $|-1, \alpha_{\downarrow}\rangle$, are not actively driven by the MW excitation and do not have coherent exchanges with the other three states.

The reconstruction algorithm is based on mapping the four fluorescence measurements to the three eigenstate populations. We define these two sets with corresponding vectors

$$V_{\text{Fluor}} = \begin{pmatrix} 1 \\ \frac{\text{Seq}_{\#3}}{\text{Seq}_{\#1}} \\ \frac{\text{Seq}_{\#4}}{\text{Seq}_{\#1}} \end{pmatrix}, \; V_{\text{States}} = \begin{pmatrix} P_{|0,\beta_{\downarrow}\rangle} \\ P_{|0,\beta_{\uparrow}\rangle} \\ P_{|-1,\alpha_{\uparrow}\rangle} \end{pmatrix}, \tag{17}$$

where $\text{Seq}_{\#}$ is the fluorescence level at the end of the relevant sequence (see Supplementary Fig. 5 for their numbering). The mapping of state populations to fluorescence levels is then implemented by the transformation matrix M:

$$V_{\text{Fluor}} = M \cdot V_{\text{States}}, \quad M = \begin{pmatrix} 1 & 1 & 1 \\ 1 & 1 & X \\ \mathcal{F} \cdot X + (1 - \mathcal{F}) & 1 & (1 - \mathcal{F}) \cdot X + \mathcal{F} \end{pmatrix}. \tag{18}$$

Here, $\mathcal{F}$ is the fidelity of the selective π-pulse, and X is the fluorescence ratio of the electronic $m_S = 0$ and the $m_S = -1$ states, calibrated by the second sequence:

$$X = \frac{2 \cdot \left(\frac{\text{Seq}_{\#2}}{\text{Seq}_{\#1}} - 1 \right)}{\mathcal{F}} + 1. \tag{19}$$

The eigenstate populations V_{States} are thus calculated by inverting the matrix M,

$$V_{\text{States}} = M^{-1} \cdot V_{\text{Fluor}}. \tag{20}$$

An example of the raw fluorescence data arising from the four sequences in Supplementary Fig. 5 -leading to the eigenstate population results of Fig. 2a in the main text- is given in Supplementary Fig. 6, where $\mathcal{F} \approx 0.75$. The eigenstate populations are then compared to a master equation simulation (described in Methods: System Hamiltonian) with the same physical parameters of the experiment (Fig. 2). The quantum master equation involving the total 6-level model of a single NV center coupled to a single ^{13}C nuclear spin is given by

$$\frac{d}{dt}\rho = -\frac{\mathrm{i}}{\hbar}[H, \rho] + \hat{\mathcal{L}}\rho, \tag{21}$$

where $\hat{\mathcal{L}}$ is the Lindblad super-operator describing laser pumping, and H is the Hamiltonian of Supplementary Eq. (1) with the HF interaction of Supplementary Eq. (16). The pumping process was modeled to reflect the incoherent transition from the $m_s = \pm 1$ states to the $m_s = 0$ state

$$\hat{\mathcal{L}}\rho = C_0 \rho C_0^\dagger - \frac{1}{2} C_0^\dagger C_0 \rho - \frac{1}{2} \rho C_0^\dagger C_0, \tag{22}$$

where $C_0 = \gamma_0 |0\rangle \langle \pm 1|$ using the experimentally determined pumping rate $\gamma_0 = 1/3\mu\mathrm{s}^{-1}$. The nuclear polarization of Fig. 2e,f is the calculated from this simulated density matrix.

Supplementary Note 3

Microwave excitation considerations for the single nitrogen-vacancy center experiments. Every NV center is comprised of a nitrogen atom and an adjacent vacancy, where the former has a nuclear spin that the electronic spin interacts with. The nuclear spin of the NV nitrogen atom is either ^{14}N or ^{15}N, with natural abundances of 99.6 % and 0.4 %, and nuclear spins $S = 1$ or $S = 1/2$, respectively. The single NV center appearing in the study had a ^{14}N, therefore every transition was split to three according to the hyperfine splitting, measured in Ref. [7] to be $A_{zz}^{^{14}\mathrm{N}} = 2.16 \pm 0.01$ MHz. The nuclear spin of the ^{14}N is not involved in the polarization process. Since the ^{14}N nuclear spin was unpolarized, a weak MW excitation ($\Omega \lesssim A_{zz}^{^{14}\mathrm{N}}$) will excite a single ^{14}N projection, while a strong MW excitation ($\Omega \gg A_{zz}^{^{14}\mathrm{N}}$) will excite the three ^{14}N projection. To optimize the weak-MW power scenario, the MW carrier was mixed with a sum of three harmonic signals with frequency difference matching the hyperfine splitting of the ^{14}N. The mixing effectively created three carrier frequencies exciting the three transitions frequencies induced by the interaction with the ^{14}N. At high MW powers, as in the Λ-regime, the mixing is not required, and the excitation was done with a single carrier frequency. During the readout phase (see Supplementary Fig. 5) the π-pulse was selective, and thus the frequency mixing was applied.

Supplementary Note 4

A. Nuclear polarization dynamics for the single nitrogen-vacancy center experiments

The polarization of the nuclear spin at a laser power corresponding to a 3 μsec electronic pumping time, was studied for the selective $\Omega \ll \delta$ regime. In Supplementary Fig. 7, we plot the nuclear polarization dynamics, as revealed by scanning the difference between the detection sequences #4 and #2 of Supplementary Fig. 5. The curves appearing in Supplementary Fig. 7 correspond to two MW frequencies resonant with the $|0, \beta_\downarrow\rangle \rightarrow |0, \alpha_\uparrow\rangle$ (orange) and the $|0, \beta_\uparrow\rangle \rightarrow |0, \alpha_\uparrow\rangle$ (blue) transitions. The time scale of the nuclear polarization transfer is approximately 10 μsec, which is a factor $\approx$3 longer than the electronic pumping time scale. The 10 μsec time scale calibrated here is relevant for all the single center measurements appearing in this study; from this, it was determined that 30 μsec of nuclear polarization build-up was sufficient to reach a steady-state.

B. Nuclear polarization dependence on the laser power

The optical pumping has two counteracting contributions to the nuclear polarization process. The first is the electronic pumping, which is essential for achieving nuclear hyperpolarization. At low laser power, the electronic polarization build-up proceeds too slowly to efficiently polarize the nuclear spin. Increasing the laser power enhances the electronic polarization rate and increases the nuclear polarization efficiency. A second competing process where the laser induces nuclear depolarization, however, arises due to the difference between the hyperfine tensor in the excited and ground electronic states. This depolarization process was experimentally seen in Ref. [1], and more thoroughly studied in Ref. [2]. Indeed, at high laser powers, the NV center has a large probability of inhabiting the excited state, leading to an acceleration of the nuclear spin depolarization because the dephasing of the nuclear state induced by the different hyperfine tensor. A laser power scan for low MW power (within the selective regime) was thus performed for a single NV center, while monitoring a first-shell ^{13}C (Supplementary Fig. 8). As expected, at low laser powers, the NV center is not efficiently polarized and thus neither is the nuclear spin. At the other end, for high laser powers, the nuclear spin depolarization is very rapid and the nuclear polarization

drops practically to zero.

C. Dependence on microwave power

In the main text, we studied the dependence of the bulk nuclear spin polarization as a function of the microwave irradiation power. Here we discuss the qualitative behavior of the nuclear polarization versus the MW power. At low MW powers, below one over the coherence rate, the efficiency of the population transfer by the MW is low, and increasing the MW power will increase the polarization transfer efficiency. At high MW powers, above Δ, the Λ-regime is reached, and the nuclear polarization is dependent on the efficiency of the transfer between the dark state $|0, \alpha_{\downarrow}\rangle$ and the bright state $|0, \alpha_{\uparrow}\rangle$. When the MW power is increased the MW radiation dresses the eigenstates and reduces the efficiency of the latter rotation, thereby reducing the nuclear polarization, even for MW powers below the broadband regime. Therefore, a clear optimum for nuclear polarization versus MW power is expected, as witnessed on Fig. 4b of the main text.

Supplementary Note 5

Estimating the number of polarized ^{13}C spins per nitrogen-vacancy center. In the following, we employ a simple phenomenological model to estimate the average number of ^{13}C spins polarized by a single NV center and the spatial distribution of the polarized ^{13}C spins. To this effect, we introduce polarization domains (spheres) containing ^{13}C nuclei around statistically distributed NV centers in the diamond crystal. All estimations are based on a diamond with an assumed NV center concentration of $c_{\mathrm{NV}} \sim 5$ ppm. With this assumption the average distance between NV centers can be calculated by

$$r_{\mathrm{avg}} = \left(N_{\mathrm{A}} \cdot \frac{\rho_{\mathrm{diamond}}}{M_{\mathrm{diamond}}} \cdot c_{\mathrm{NV}}\right)^{-\frac{1}{3}} = \left(6.023 \cdot 10^{23}\,\mathrm{mol}^{-1} \cdot \frac{3.52\,\mathrm{g\,cm}^{-3}}{12.01\,\mathrm{g\,mol}^{-1}} \cdot 5 \cdot 10^{-6}\right)^{-\frac{1}{3}} \tag{23}$$

$$= 100.4\,\text{Å}, \tag{24}$$

where N_{A} is the Avogadro constant and ρ_{diamond} and M_{diamond} are the density and the molar mass of diamond. In the special case of polarizing the aligned orientation (Fig. 3c in the main text) only one crystallographic direction, corresponding to 25% of the NV centers, contributes to the electron-nuclei polarization transfer process, increasing the average distance to $\sim$ 165.5 Å. By neglecting other crystal defects the polarization domain of a single NV defect is simplified to a sphere with a radius of $r = 83$ Å.

The Boltzmann distribution of a statistical ensemble of ^{13}C spins at 4.7 T in a fully-relaxed state corresponds to a population difference between the energy levels of $\sim$8 ppm (for each million of spins there are 8 spins more to be found in the lower energy level). The experimentally observed enhancement factors of up to $\sim \pm 250$ are equivalent to population differences of $\sim \pm 1600$ ppm. In other words for each million spins there are up to 1600 spins more to be found in the lower/higher energy level. The diamond lattice contains $n_{\mathrm{unit}} = 8$ atoms per unit cell with a lattice parameter of $a = 3.57$ Å at room temperature. The numbers of ^{13}C atoms in a sphere with an assumed radius $r \sim 83$ Å can be approximated by

$$n_{^{13}C} = \frac{n_{\mathrm{unit}}}{a^3} \cdot \frac{4}{3}\pi r^3 c_{^{13}C} \approx 42000 \tag{25}$$

where $c_{^{13}C}$=0.1 is the 10% enriched abundance of ^{13}C spins. This means that a number of ^{13}C spin flipped by each NV center is in the order of 70. While this back-of-the-envelope calculation is sufficient to give a rough estimation of the polarized spins per the NV domain sphere, we would like to emphasize the approximative and phenomenological nature of this

derivation. In addition, the number of spin flips is an average number. It is plausible to expect much higher local enhancement factors around the NV centers, while the contribution of the majority of the ^{13}C spins in the remainder of the NV sphere to the macroscopic polarization, is probably much lower.

Supplementary References

[1] Dutt, M. V. G. *et al.* Quantum register based on individual electronic and nuclear spin qubits in diamond. *Science* **316**, 1312–1316 (2007).

[2] Jiang, L. *et al.* Coherence of an Optically Illuminated Single Nuclear Spin Qubit. *Phys. Rev. Lett.* **100**, 073001 (2008).

[3] Jacques, V. *et al.* Dynamic polarization of single nuclear spins by optical pumping of nitrogen-vacancy color centers in diamond at room temperature. *Phys. Rev. Lett.* **102**, 057403 (2009).

[4] Fischer, R., Bretschneider, C. O., London P., Budker, D., Gershoni, D. & Frydman, L. Bulk nuclear polarization enhanced at room temperature by optical pumping. *Phys. Rev. Lett.* **111**, 057601 (2013).

[5] Slichter, C. *Principles of magnetic resonance* (Springer, 1996).

[6] Shim, J. H. *et al.* Characterization of hyperfine interaction between single electron and single nuclear spins in diamond assisted by quantum beat from the nuclear spin. Preprint at http://arxiv.org/abs/1307.0257 (2013).

[7] Steiner, M., Neumann, P., Beck, J., Jelezko, F. & Wrachtrup, J. Universal enhancement of the optical readout fidelity of single electron spins at nitrogen-vacancy centers in diamond. *Phys. Rev. B* **81**, 035205 (2010).
